# General review of titanium toxicity

**DOI:** 10.1186/s40729-019-0162-x

**Published:** 2019-03-11

**Authors:** Kyeong Tae Kim, Mi Young Eo, Truc Thi Hoang Nguyen, Soung Min Kim

**Affiliations:** 1 Department of Dentistry, Dental Research Institute, Seoul National University, Seoul, South Korea; 2 Department of Oral and Maxillofacial Surgery, Dental Research Institute, School of Dentistry, Seoul National University, Seoul, South Korea; 3 Oral and Maxillofacial Microvascular Reconstruction LAB, Ghana Health Service, Regional Hospital, P.O. Box 27, Sunyani, Brong Ahafo Ghana

**Keywords:** Titanium toxicity, Titanium dental implant toxicity, Titanium allergy, Titanium corrosion, Yellow nail syndrome

## Abstract

**Background:**

Titanium is a commonly used inert bio-implant material within the medical and dental fields. Although the use of titanium is thought to be safe with a high success rate, in some cases, there are rare reports of problems caused by titanium. In most of these problematic reports, only individual reports are dominant and comprehensive reporting has not been performed. This comprehensive article has been prepared to review the toxicity of titanium materials within the medical and dental fields.

**Methods:**

We used online searching tools including MEDLINE (PubMed), Embase, Cochrane Library, and Google Scholar by combining keywords such as “titanium implant toxicity,” “titanium implant corrosion,” “titanium implant allergy,” and “yellow nail syndrome.” Recently updated data has been collected and compiled into one of four categories: “the toxicity of titanium,” “the toxicity of titanium alloys,” “the toxicity of titanium implants,” and “diseases related to titanium.”

**Results:**

Recent studies with regard to titanium toxicity have been increasing and have now expanded to the medical field in addition to the fields of environmental research and basic science. Problems that may arise in titanium-based dental implants include the generation of titanium and titanium alloy particles and ions deposited into surrounding tissues due to the corrosion and wear of implants, resulting in bone loss due to inflammatory reactions, which may lead to osseointegration failure of the dental implant. These titanium ions and particles are systemically deposited and can lead to toxic reactions in other tissues such as yellow nail syndrome. Additionally, implant failure and allergic reactions can occur due to hypersensitivity reactions. Zirconia implants can be considered as an alternative; however, limitations still exist due to a lack of long-term clinical data.

**Conclusions:**

Clinicians should pay attention to the use of titanium dental implants and need to be aware of the problems that may arise from the use of titanium implants and should be able to diagnose them, in spite of very rare occurrence. Within the limitation of this study, it was suggested that we should be aware the rare problems of titanium toxicity.

## Background

Titanium is one of the most widely used materials for dental implants due to its mechanical strength, biocompatibility, and a long history of use [1, 2]. Current titanium dental implants possess a high success rate; however, failures are still being reported [3–5]. Cause of these implant failures can be poor oral hygiene, uncontrolled deposition of plaque, and calculus around the implant which cause peri-implantitis or occlusal problems. In the light of new investigations in biological and mechanical aspects, the allergy response to dental implant materials and toxicity of the particle released from implant system are reported to have a role in implant failure [6, 7]. There are also a variety studies on titanium and its alloys as well as implant surface treatment materials to determine their toxicity behavior and its mechanism [8, 9]. Typical examples include bone loss due to inflammation reactions due to implant corrosion [10–12], hypersensitivity to titanium and allergic reactions [13–16], and yellow nail syndrome [17–20].

Titanium is also used commonly in industrial applications such as coatings for pharmaceuticals, processing materials for gum and confections, food additives, and paints. In the medical field, titanium and titanium alloys have been used to fabricate various of implantation and fixation systems. With the widespread use of titanium, there are concerns regarding the adverse effects of titanium accumulation and its effects on the human body [21, 22]. Therefore, stability and potential hazards of titanium should also be evaluated and discussed.

The purpose of this article is to provide a general overview of the stability and risk associated with titanium materials and to suggest alternative solutions. We examined the toxicity of titanium through a division into four categories: the toxicity of titanium, the toxicity of titanium alloys, the toxicity of titanium implants, and diseases related to titanium.

## Methods

### Focus question

“What is the general overview of the risks and stability associated with titanium materials?”

### Literature search

This review was prepared using data collected from until November 2018 through a keyword online search using MEDLINE (PubMed), Embase, Cochrane Library, and Google Scholar. Additional data were gathered for the necessary detailed parts using keywords including titanium toxicity, titanium alloy toxicity, titanium implant toxicity, and yellow nail syndrome; a total of 4213 articles were searched.

### Inclusion criteria

We searched the toxicity of titanium through a division into four categories: the toxicity of titanium, the toxicity of titanium alloys, the toxicity of titanium implants, and diseases related to titanium.

### Exclusion criteria

The exclusion criteria were as follows: non-English publications and duplicated articles.

### Screening

In order to investigate the relevance of the main topic with recent studies, we collected the data from 1991 to November 2018 and keywords were also limited to “titanium toxicity human,” “titanium alloy toxicity human,” “titanium implant toxicity,” “titanium corrosion,” and “yellow nail syndrome titanium”; thus, 1820 articles were found. Additionally, another 464 articles that were found from the “titanium implant corrosion” keyword and another 84 articles that were found from the “titanium implant allergy” keyword were further identified and included in the “toxicity of titanium implant” category.

### Data extraction

Based on these data, we divided the summarized data into one of four categories: the toxicity of titanium, the toxicity of titanium alloys, the toxicity of titanium implants, and diseases related to titanium.

## Results

Extensive data was searched as mentioned in the research methods. According to the data analysis, the number of papers from 2011 to 2015 was the highest at 730; the research shows a trend of rapid increase in recent years with the large number of papers from 2016 to 2018. In the author’s field of specialization, 995 fields of basic science were the most studied; fields within environmental science and basic science were extended to the medical field (Table 1). The toxicity of titanium implants revealed 734 results, including titanium corrosion and titanium hypersensitivity, 1229 articles for titanium toxicity, 149 toxicities for titanium alloy, and 256 articles for yellow nail syndrome, a titanium-related disease (Table 2).

**Table 1:** Number of articles representing each titanium toxicity trend according to year

	1991–1995	1996–2000	2001–2005	2006–2010	2011–2015	2016–2018
Titanium toxicity	30	58	81	216	432	412
Titanium alloy toxicity	12	17	30	18	40	32
Titanium implant toxicity	8	20	30	26	53	55
Titanium implant corrosion	20	48	59	88	122	120
Titanium allergy	2	5	5	21	22	30
Yellow nail syndrome	22	30	31	39	61	73
Total	94	178	236	408	730	722

**Table 2:** Specialized scope of published articles with regard to titanium toxicity

	Dental	Orthopedic	Medical	Environment	Basic
Titanium toxicity	82	102	150	252	643
Titanium alloy toxicity	42	30	35	5	37
Titanium implant toxicity	37	56	22	3	74
Titanium implant corrosion	145	63	22	7	220
Titanium allergy	22	26	20	2	15
Yellow nail syndrome	–	–	250	–	6
Total	328	277	499	269	995

Within the “Toxicity of titanium” section, studies with regard to liver, lung, and kidney cytotoxicity in human cells and the accumulation of titanium particles were summarized. Within the “Toxicity of titanium alloys” section, we summarized the cytotoxicity of titanium alloys and the inflammatory response of surrounding tissues caused by titanium alloys. Within the “Toxicity of titanium implants” section, we reviewed the inflammatory response to titanium corrosion, hypersensitivity to titanium, and the potential risks of nanoparticles used in titanium implants. Finally, we reviewed the toxicity of titanium by surveying yellow nail syndrome as a disease related to titanium and discussed the risks and potential risks of titanium.

### Toxicity of titanium

Titanium is used in various applications such as cosmetics, paints, food products, drugs, and medical implant materials including dental implant [22, 23]. Currently, the most commonly used form of titanium is TiO_2_ powder. As the production of TiO_2_ powder continues to expand, there has been an increase in concern of its influence on human and environment [24, 25]. Numerous studies reported the presence and toxicity of TiO_2_ nanoparticle in both animal models and cultured human cell.

The toxicity of TiO_2_ nanoparticles (TiO_2_-NP) in rodents has been reported. Many authors studied the serum biochemical parameters, pathology changes, and the biodistribution of TiO_2_-NP in the liver, kidneys, lung, spleen, and brain tissue by facilitating a variety of methods including blood biomarker assays, histopathological examination, etc. The dependence of experiment results on the intake (inhalation, oral administration, intraperitioneal/intravenous injection), dosages, and different sizes of nanoparticles was also discussed [21, 26–33].

In two studies regarding the pulmonary response of rodents to subchronic inhalation of TiO_2_-NP, Bermudez et al. reported a dose-dependent expression of lung burdens in mice, rats, and hamsters in exposure to a wide range of TiO_2_ pigment. Rats also developed a unique progressive fibro-proliferative lesions alveolar epithelial metaplasia in response to high dose of TiO_2_-NP [21, 31]. Warheit et al. also reported the species-specific keratin cysts in rats under the overload exposure condition [30].

The acute toxicity and biodistribution were discussed in Wang et al., Chen et al., and Fabian et al. studies [26–28]. Wang et al. [26] reported the injury in the liver (hydropic degeneration around the central vein in the liver and spotty necrosis of the hepatocyte) and kidneys (the BUN level was increased with pathologic renal changes) after oral administration with large dose (5 g/kg body) of different sizes of TiO_2_ particles (25, 80, and 155 nm). The biodistribution examinations also showed predominant accumulation in the liver, kidney, spleen, and lungs of TiO_2_, which indicated the ability of TiO_2_ be transported to other organs after oral intake. Following this report, Chen et al. [27] also reported the pathological changes of the spleen, heart, liver, lung, and kidneys caused by acute toxicity in rats injected with TiO_2_-NP. The influence of TiO_2_ on the central nervous system (CNS) is gaining attention recently. Valentini et al. investigated the toxicity of TiO_2_-NP to the cortical neuron cultures and in the brain of rats, reported the clear impact of TiO_2_-NP on the neuronal cells and rat brain, and indicated the new evidences of TiO_2_-NP toxicity in CNS [32].

While there have been reports of titanium toxicity in animal models, Fabian et al. and Warheit et al. [28, 34] reported that the risk of titanium toxicity was not significantly high. In a low-dose TiO_2_ experiment, Fabian et al. [28] reported no obvious toxic health effects and no detectable inflammatory response or organ toxicity in the rats intravenously injected with suspension of TiO_2_ in serum (5 mg/kg body weight), despite of the expected biodistribution. In addition, Warheit et al. introduced ten different toxicity studies to form a base set of hazard test for TiO_2_ ultrafine particle and found that most of the studies indicated low hazard potential in mammals or aquatic species [34].

The effects of TiO_2_ nanoparticle toxicity in the cultured human cell were also studied [35–39]. There have been a variety of human cell lines used in TiO_2_ toxicity assessment experiments, including mesothelial cell, epithelial cell, trophoblast cell, and lymphoblastoid cell. In vitro studies reported by Wang et al. showed UF-TiO_2_ genotoxicity and cytotoxicity in human lymphoblastic cells, with the induction of apoptosis following exposure to UF-TiO_2_ [35]. Kuku and Culha used surface-enhanced Raman spectroscopy (SERS) for the multidimensional cellular dynamic information to exam the toxic response to TiO_2_-NP of three cell lines of vein (HUVEC), lung carcinoma (A549), and fibroblast in skin (L929). The results pointed out that L929 is the most resistant cell line, while the HUVEC and A549 cell lines showed the collagen and lipid deformative phenomenon, respectively [36]. Even though the pathological changes such as apoptosis and fibro-proliferative expression of the epithelial cells have been studied in several animal models, its precise mechanism is still not determined. Recently, Kim et al. reported an in vitro experiment regarding the expression of mucin genes in human airway epithelial cells. The authors confirmed that TiO_2_-NP initiated the TLR4-dependent pathway, leading to the MUC5B overproduction, which relates to the inflammatory response in human airway [37]. In Suarez-Lopez del Amo et al. experiment, the TiO_2_ particles derived following implantation were collected and co-cultured with the oral epithelial cells (NOK-SI). Two markers DDR and BRCA1 were used to detect DNA damage repair. The authors suggested that compared to DDR, BRCA1 is an optimal marker for detecting DNA damage induced by Ti particles [38].

Ti implants are always inserted into diverse complex body environments which contain various inorganic and organic molecules, as well as living cells. Therefore, besides the behavior of Ti particles in animal body and cell culture, the influence of serum proteins or other biomolecules on titanium implant has been studied under different experimental conditions. Jackson et al. studied the absorption behaviors of bovine fibrinogen and bovine serum albumin (BSA) at the commercially pure titanium surface [40]. The releasing of titanium particles into surrounding tissue by protein adsorption and subsequent desorption of formed metal-protein complexes can cause varieties of tissue reactions. It has been also demonstrated that some conditions such as inflammation or formation of microbial biofilm can lead to a locally acidified environment, and this environment can be potentially harmful to titanium implant. Yu et al. gave attention to lipopolysaccharide (LPS) due to its crucial role as a mediator in peri-implant inflammation. The study demonstrated that LPS significantly inhibited Ti release under the low acidic conditions (pH = 2) but promoted Ti release at the mildly acidic and neutral pH levels, which supposed to be encountered in the peri-implant environment [41].

To approach the mechanism of body reaction to TiO_2_-NP, many studies reported inflammatory effects due to TiO_2_-NP exposure, including the presence of pro-inflammatory mediators, macrophage inflammatory proteins, and other inflammatory molecules [42]. The interaction between TiO_2_-NP and inflammatory cytokines, including CXCL8, a clinically relevant pro-inflammatory chemokine, was also investigated by Batt et al. [43]. The authors found that the TiO_2_-NP could preferentially adsorb CXCL8 (and IFN-γ), which leads to the disruption of neutrophil chemotaxis and modifies local inflammatory mediator concentration and might result in hampered inflammatory response.

The potential risks of TiO2 accumulation in the body have been followed by reports of metal debris from titanium prosthesis wear. According to Engh et al.’s report [44], the accumulation of metal debris such as titanium, aluminum, and vanadium was found in the bone marrow of two patients who had implanted joints in the iliac. One of the two patients was diagnosed with leukocytopenia, anemia, and general weakness. Although it is questionable that whether these conditions were due to an accumulation of titanium toxicity or not, attention needs to be paid to the fact that metal debris from worn out implants can accumulate in the liver, spleen, and bone marrow, causing adverse effects on the body and systemic disease.

### Toxicity of titanium alloy

Titanium alloys have many applications in medical implantation, including orthopedic prostheses and dental implant. Various studies have been conducted regarding the effects of metal particles which worn out from orthopedic prostheses [45]. In 1981, Rae [46] performed experiments in which human synovial fibroblasts were exposed to various preparations of metals and alloy, including pure titanium and wear debris from titanium alloy (Ti-6Al-4V). In the experiment conditions, fibroblasts incubated with pure titanium and titanium alloy showed no significant increases in lactate dehydrogenase and no morphological change. Besides, due to the high solubility of vanadium in the cultured cells, the author estimated that the vanadium from titanium alloy might be potentially harmful to the cell.

In 1993, Haynes et al. [47] performed experiments using titanium-aluminum-vanadium (Ti-Al-V) and cobalt-chromium (Co-Cr) particles of similar size range and concentration similar to those found in failed hip prostheses. In the abdominal macrophage experiments of rats, Co-Cr yielded a high toxic response while Ti-Al-V increased the release of inflammation-inducing mediators such as prostaglandin E2, interleukin-1, interleukin-6, and tumor necrosis factor. These results implied that debris particles of worn Ti-Al-V could induce the release of inflammatory mediators affecting the tissues surrounding the prosthesis and cause osteolysis. Rogers et al. [48] tested the toxicity of vanadium and niobium in titanium alloys; human monocytes released more inflammatory mediators due to Ti-Al-V compared to titanium-aluminum-niobium (Ti-Al-Nb). The authors thus suggested that metal debris particles might lead to bone loss around the prosthesis.

Studies regarding titanium alloy toxicity were continuously reported in 2000s. Hallab et al. [49] performed experiments using human lymphocytes. Co-Cr-Mo and Ti-6Al-4V were incubated with human serum. This experiment showed that a complex between the protein and metal particles caused a lymphocyte reaction; protein binding with a higher molecular weight caused a larger inflammatory reaction. Dalal et al. [50] experimented with the influence of metal particles in human peri-implant cells, osteoblasts, fibroblasts, and macrophages. Co-Cr-Mo, titanium alloy, zirconium oxide, and zirconium alloy were used. Co-Cr-Mo yielded a toxic reaction that interfered with the viability and proliferation of osteoblasts, fibroblasts, and macrophages. All particles induced inflammatory mediator release to macrophages; Co-Cr-Mo, a titanium alloy, released more inflammatory mediators. These results showed that particles around the metal prosthesis could cause irritation and lead to the failure of orthopedic prostheses.

The behavior of titanium alloy in body environment is affected by complex factors. Yu et al. investigated the synergistic effect of albumin and H_2_O_2_ on corrosion of Ti6Al4V in physiological saline. In the presence of both H_2_O_2_ and albumin, there was a very much higher rate of metal release from Ti6Al4V compared to the presence of albumin and H_2_O_2_ alone [51]. Furthermore, in a recent study, Zhang et al. continuously worked on the synergistic effect of albumin and H_2_O_2_ on corrosion of Ti6Al4V in physiological saline with electrochemical method and showed the time-dependent dissolution of Ti6Al4V [52]. The experiment showed that albumin suppressed the dissolution in the presence of H_2_O_2_ at short periods (< 24 h), but over longer time periods, the dissolution rate increased, which might be attributed to the reduction of oxide film due to albumin-catalyzed dissolution of H_2_O_2_ corrosion products. The authors emphasized the importance of a realistic solution and a longer time period experiment design in testing corrosion resistance of metallic biomaterials.

In dental titanium implants, it was suggested that implantation failures may be caused by inflammatory reactions in surrounding tissues due to titanium alloy corrosion or the allergic reaction to titanium and titanium alloys [2, 3]. This topic will be discussed below.

### Toxicity of titanium implants

According to the American Society for Testing and Materials (ASTM), there are four grades of commercially pure titanium (CpTi) used in implant biomaterial. The grades I–IV CpTi have different purity grades, with different amounts of interstitial elements (carbon, oxygen, nitrogen, hydrogen, and iron). The grade V refers to the titanium alloys Ti-6Al-4V, which is the most commonly used alloy. Besides, currently, a variety of nanometerials are used for the surface treatment of titanium-based dental implants. Among those coating material, two titanium-contained coating materials are Ti and TiN (Titanium Nitride) which have been studied and advocated to improve the chemical and wear resistance of titanium implant [53]. Even though titanium and its alloys are considered as the most biocompatible implant material because of their nobly biochemical characteristic, wear and corrosion still occur especially in an extreme environment like oral. The released particle can come from the titanium coating layer or from the titanium implant itself. Both phenomena have been studied and reported in many articles and will be reported separately.

Maritini et al. compared implantation of titanium powder plasma-spray-coated titanium screws (TPS-Ti) and fluorohydroxyapatite-coated titanium screws (FHA-Ti). Authors reported the evidence of titanium dispersion inside the medullary spaces when TPS-Ti was implanted, which was the result of friction occurring at the implant surface-host bone interface, leading to loss of integration in the coating layer and release of the detachment of metal particles to surrounding tissue [54].

It is well established that titanium dioxide forms and covers the surface of implant, which makes it highly resistant to corrosion. However, in recent studies, particles of implants were found in peri-implant tissues, which may strongly suggest that a corrosive process has occurred on the titanium implant. The effect of different environmental factors on dental implants was also studied. There are reports that corrosion is significant in conditions which have low pH or high concentration of fluoride [55–58]. In an in vitro study by Strietzel et al. [55], influence of the presence of fluorine on titanium corrosion was detected. Corrosion is further enhanced at lower pH and less influenced by organic acids and their pH values. Schiff et al. [57] tested the effects of fluorine and pH on titanium and titanium alloys and found that fluorine ions could destroy and corrode titanium and titanium passivation layer. The titanium alloys that were used were Ni-Ti, Ni-Ti-Co, and TiAl6V4. Furthermore, in a recent study, Penarrieto-Juanito et al. evaluated ion releases from dental implant systems in fluoride and hydrogen peroxide and examined the surface changes in this process. SEM images indicated the excessive oxidation in implant-abutment joint surfaces along with releasing of Ti, Al, and V ions after being immersed in 1.23% sodium fluoride gel, while no significant corrosion was observed in hydrogen peroxide environment [58].

Recently, there are more studies working on the linking of titanium implants and implant complication or failure. Wachi et al. reported that Ti ions may be involved in the deteriorating effects of peri-implant mucositis, which can develop into peri-implantitis accompanied by alveolar bone resorption [59]. Olmedo et al. reported two cases of reactive lesions of peri-implant mucosa associated with titanium dental implants, one case was diagnosed as pyogenic granuloma and the other case as peripheral giant cell granuloma. The presence of metal-like particles in the tissues suggests that the etiology of the lesions might be related to the corrosion process of the metal structure. In a previous study, the authors found macrophages loaded with titanium particles as indicators of the corrosion process in the soft peri-implant tissue of failed human dental implants [60].

Assuming the implant particle can be an initiator of the peri-implantitis, many studies have been performed to approach the oral mucosa tissue’s response to titanium implant as well as implant cover screws. To determine the correlation between titanium particles and peri-implantitis, Olmedo et al. performed the exfoliative cytological test and observed particles inside and outside the epithelial cells and macrophages. Also, the experiment pointed that the concentration of implant particles in the peri-implantitis group was significantly higher than in the control group [61]. The recent study of Penmetsa et al., in which the exfoliative cytology was also used to detect the titanium particle in a group which has mild gingivitis and another group which has moderate-to-severe condition, also has the similar result. Sixty percent of the specimens in the moderate-to-severe group had titanium particles in peri-implant cytology [62].

In Wilson et al. study, 34 among a total of 36 human peri-implantitis biopsies were analyzed [63]. The SEM images revealed the predominant titanium particle surrounded by inflammatory cells. The study mentions three possibilities that can cause the presence of titanium particle in surrounding tissue. They are the releasing due to the friction between implant and bone surface during installation, the wear during debridement at maintenance visits, and the corrosion [63]. Fretwurst et al. reported the metal particle in peri-implant soft tissue along with M1 macrophages and the increasing in titanium concentration with lymphocytes detection [64]. In association with the metal particle releasing, the damage of implant surface during the installation procedure was also determined [65].

On the other hand, a study of Addison et al. using synchrotron X-ray microfocus spectroscopy in order to detect trace distribution of Ti in tissue demonstrated a scattered and heterogeneous distribution of Ti in inflamed tissues taken from around skin-penetrating Ti implants. The location and distribution characteristics of Ti particles suggested that debris from implant placement are unlikely to be the major contributors. The authors proposed that Ti in the tissue results can be derived from micro-motion and localized corrosion in surface crevices [66].

One of the causes of implant failure can be attributed to allergic reactions to titanium. There have been reports of hypersensitive reactions such as erythema, urticaria, eczema, swelling, pain, necrosis, and bone loss due to titanium dental implants [15, 67, 68]. Despite of the limitation of the case report, these cannot be neglected. In several case reports in which titanium allergy was initially suspected, upon further investigations, the allergic agents were other metals [69]. The reliability of the patch test for current titanium is not guaranteed for clinical use. Therefore, it seems that future studies and countermeasures are necessary [70].

The case for allergies after installation of titanium dental implants was recorded by Hosoki et al. [14] at a 69-year-old male. The patient had the successful dental implantation in 2008. An allergic eczema reaction occurred in 2010 after inserting of a titanium screw due to a leg fracture. The titanium screw was removed a year later; however, the eczema was only reduced by 50%. All metal prostheses except the implant screw and abutment were removed, and the eczema reaction was reduced to 30%; the symptoms still remained. The removal of the titanium implant screw and abutment in 2014 led to a full recovery. In Korea, allergy condition has also been reported after the installation of titanium implants [15]. In 2012, a 70-year-old woman exhibited a stomatitis that appeared to be an allergic reaction. There was no evidence of metal hypersensitivity in this patient. There were no problems with the implant placement; however, after the abutment was raised and the prosthesis was made, the patient complained of pain. Removal of the prosthesis confirmed erythema on the gingiva around the abdominal cavity. Allergic symptoms were suspected to be due to TiN-coated abutments, and symptoms improved after the use of titanium abutments. A patch test showed a positive result for TiN.

Allergic reactions to titanium materials have also been reported with orthopedic prostheses. Thomas et al. [71] reported eczema symptoms and improper bone formation in the case of a 35-year-old male patient with a titanium implant in the fracture of his hand. In this case, the patch test showed a negative reaction to titanium, nickel, chromium, cobalt, etc. However, the lymphocyte transformation test showed an increased pattern for titanium. Additionally, in 1991, Lalor et al. also reported hypersensitivity reactions to titanium and reported the proliferation of inflammatory cells in patients with failed orthopedic prostheses [72].

Although the biocompatibility of titanium has been evaluated to be good because it causes less hypersensitive reaction than other metals, it does not mean that allergy symptoms related to titanium do not exist. Previous reports have shown that hypersensitive reactions to titanium and titanium alloys can lead to failures in clinical treatment. Therefore, allergy symptoms of titanium or titanium alloy components should also be accounted for a related factor to dental implants failures.

### Titanium-related diseases

It has been reported that systemic disease can occur due to titanium. According to a study by Berglund and Carlmark [17] in 2011, titanium can be attributed to the cause of “yellow nail syndrome.” In 30 patients with yellow nail syndrome, energy-dispersive X-ray fluorescence (EDXRF) was used to measure the titanium content in the nails of patients; the titanium content was found to be high, and titanium was identified as the cause of yellow nail syndrome. Yellow nail syndrome is characterized by a change in the nails, bronchial obstruction, and lymphedema. Berglund and Carlmark also reported that postnasal drip and cough-associated sinusitis are the most common symptoms found in yellow nail syndrome patients.

Yellow nail syndrome was first designated as a medical term by Samman and White [73] during their report of a patient with nails growing slowly, thicker, and yellowish in color in conjunction with lymphedema syndrome. These cases also reported recurrent pleural effusion, intermittent coughing with bronchial asthma accompanied by sputum, bronchiectasis [74–76], and inflammation in the maxillary sinus and sinus [77–81]. In 1994, Varney et al. [79] reported 17 patients with yellow nail syndrome. Among that, 14 patients had rhinosinusitis (83%) and had daily mucopurulent rhinorhoea and nasal obstruction. The onset of nasal symptoms could predate nail change or appear at the same time. Additionally, in 2014, Piraccini et al. [81] reported that the mean patient age was 57 years in a report of 21 patients; most patients had a history of pathology in which 16 patients experienced chronic respiratory disease and six patients had lymphadenopathy. A change in nail color appeared to be a symptom that was revealed after progression of the disease and did not necessarily have to occur. Lymphedema was also seen when the disease persisted for a long time. Pleural effusion was the most common lung change, and chronic sinusitis was reported to occur with an early onset. At least 10 of 20 patients were reported to show an improvement in symptoms after 6 months of continuous vitamin E_1_ administration at 200 IU/day; however, there was a continuous debate regarding the medication details.

Efforts to elucidate the pathogenesis of yellow nail syndrome are currently underway. In 2001, D’Alessandro et al. [76] reported that the protein content of pleural effusions was high in yellow nail syndrome patients and reported the relationship between hypoalbuminemia and a reduction in systemic albumin. As mentioned above, in 2011, Berglund and Carlmark [17] evaluated 30 patients with yellow nail syndrome via EDXRF and found that titanium was detected in yellow nail syndrome. Titanium was thus judged to be a pathogen of yellow nail syndrome. The main source of titanium ions was reported to be due to corrosion caused by galvanic effects between titanium implants and gold and/or amalgam restorations and corrosion due to fluorine oxidation. In 26 patients with titanium implant, including 20 patients with titanium implants in the jaw and mouth, 20 patients with gold restorations in the mouth, 2 patients with amalgam restorations, and 2 patients with gold rings, oral galvanic action was possible. In 4 of these patients, removal of the gold restoration resulted in a recovery of the symptoms originating from galvanic action. Patients with implants with a symptomatic recovery experienced a recurrence of symptoms when later exposed to titanium again. In 3 patients, dental titanium tools were exposed to fluoride gels and fluoride solutions [17]. In some other patients, titanium dioxide contained in drugs was considered to be the source of titanium ions. Four male and 4 female patients suffered from yellow nail syndrome after eating TiO_2_-containing medication such as diclofenac, celecoxib, and zopiklon, along with gum, candy, and licorice. In this case, symptoms were remedied by not using medication [17]. Other reports showed a case of yellow nail syndrome after drug ingestion of medicine containing TiO_2_. In these reports, symptoms improved when drug usage was discontinued [17, 82].

There are numerous reports showing the association between titanium and yellow nail syndrome in addition to the above reports. In 2015, Decker et al. [19] reported a case of a 67-year-old female patient who had lost her claws 18 months prior and had changes in bronchitis, sinusitis, and nails within the last 5 years. Inhaled corticosteroids were used for initial continuous cough symptoms but were not effective. These early respiratory symptoms were followed by changes in nails 3 years later. At the same time, *Pseudomonas aeruginosa* bronchitis and sinusitis were experienced. Lymphedema was not observed, and vitamin E 1600 IU/day treatment was prescribed. EDXRF of the nails revealed high levels of titanium; eight amalgam restorations and fluoride-containing toothpastes were used daily in the oral cavity. She was also reported to have a history of regular titanium dioxide intake through cetirizine (10 mg/day) and gum (4–8 piece/day). Ataya et al. [20] reported a 56-year-old woman with appearance of yellow nail syndrome symptoms immediately after implantation. Chronic sinusitis, cough, a change in nails, and maxillary sinusitis were all recovered after implant removal. However, they reported that there was no change in the nails. This report also showed that yellow nail syndrome was associated with titanium. Dos Santos [83] also reported the association of yellow nail syndrome with titanium in 2016, and De Lima and Dos Santos et al. [84] reported the observation of titanium accumulation in the liver, spleen, lung, lymph nodes, and bone marrow in the autopsy results of five drug-addicted patients; titanium pigmentation was observed under a microscope. This report revealed a systemic accumulation of titanium, but with no change in the nails.

As in the aforementioned reviews, the accumulation of titanium has been observed in patients with “yellow nail syndrome” and the relationship is currently being discussed in greater detail. There have been several reports of the relative association between titanium and yellow nail syndrome at the beginning of Berglund and Carlmark’s report [17]; on the contrary, there was no evidence of “yellow nails” in the anatomical studies of patients who were drug addicted. This is still a controversial topic which is still in debate. Therefore, further studies are needed to determine the relationship between titanium and yellow nail syndrome and the pathogenesis.

## Discussion

Titanium is presently used in a variety of applications including dental implants, orthopedic prostheses, industrial cosmetics, drugs, confections, and paints. Due to its extensive usage, issues related to stability need to be discussed. Titanium dental implants possess many advantages and are now widely used with high rates of success. However, we need to look at the aforementioned potential risks and issues that are reported in small cases. This review article provided a comprehensive review of the potential causes of titanium failures and failures in titanium implants with a discussion of alternatives.

The first problem of titanium toxicity was related to the failure of titanium implants, which was mainly covered in the “Results” section. Titanium abrasion and corrosion are believed to cause inflammation in the surrounding tissues, which can be the initiator of peri-implantitis and lead to the failure of the implant [7]. There are reports that implant corrosion can be attributed to the cause of implant failures; reports from orthopedic surgeons have shown that particles due to implant wear have contributed to inflammation [11]. These abrasions could occur during abutment connection and disconnection, bony resorption, and intraoral use during the insertion of titanium implants. Corrosion could also occur due to fluoride and oral fluids, other restorations, and galvanic action.

Titanium materials are mainly alloyed with aluminum and vanadium. Vanadium and niobium have also showed to induce the release of inflammatory mediators; other alloy components may also be involved in the inflammatory response of titanium alloy corrosion [45]. Therefore, it is necessary to consider the influence of particles due to titanium alloy corrosion in the case of failure due to peri-implant inflammation. It is also necessary to study the method of caution and interception of galvanic action between other metal prostheses when implants are installed. Care should also be taken when using fluoride in patients.

Tribocorrosion is a relatively new research field regarding behavior of dental implant system. This is defined as a tribosystem which has three interrelated components: tribology (friction, wear, and lubrication), corrosion (material and environmental factors), and biochemistry (interactions between cells and protein) [85]. The wear and corrosion in the contacting surface of implant fixture and implant abutment can cause the failure of dental implant system, and the wear debris and metallic ions due to the tribocorrosion phenomena can become toxic for human tissues. Barbieri et al. studied the corrosion behavior of dental implant in human saliva environment and reported significant values of Ti released by micro-sanded and acid-etched dental implants immersed in human saliva [86].

The wear and corrosion interactions at the titanium-zirconia interface were discussed in a pilot study of Klotz et al. as a cause of metal releasing from dental implants [87]. The study reported that the implants with the zirconia abutments showed a greater initial rate of wear and more total wear than the implants with the titanium abutments following cyclic loading. Stimmelmayr at al. determined and measured the wear of the interface between titanium implants and one-piece zirconia abutments in comparison to titanium abutments. Titanium implants showed higher wear at the implant interface following cyclic loading when connected to one-piece zirconia implant abutments compared to titanium abutments [88].

In a recent in vitro study, Sikora et al. displayed an opposite result [89]. The in vitro study clarified the mechanical and chemical relationship that could occur between materials at the implant-abutments interface; however, zirconia abutments result in less deterioration at the implant-abutment interface, potentially leading to less metal release, less tissue damage and tattooing, and superior long-term outcomes. Future studies will explore such effects by simulating an advanced clinical setting [89].

Corrosion products released from the surfaces of dental implants can be swallowed. The absorbed titanium can accumulate in other organs in the body as shown in the animal and cultured cell experiments [90]. In addition, Feng et al. [91] reported that nanoparticles can pass through the blood-brain barrier (BBB) and may be toxic to the central nervous system (CNS). The testing methods still have limitation and further studies need to be performed, but the neurotoxic nanoparticles including titanium particles is indicated, which requires a development of safety assessment systems.

In case of the particles from corrosion process accumulate in the gingival sulcus, corrosion products can diffuse into adjacent tissues and cause the blue-gray pigmentation of gingiva and root dentin [91]. There are various studies on the tissue behavior and long-term effect related to amalgam tattoo; however, the similar studies on titanium tattoo are rare. A study on pigmentation of human teeth and gingiva evaluated the composition of common metal used in dental alloy, including titanium, as reported by Venclikova et al. Authors indicated the detection of titanium in tissue around an implant which had the presence of oral mucosa pigmentation. The titanium needle-like deposits were found in the extracellular matrix around fibroblasts and macrophages [92]. More recently, Taylor et al. report two cases of titanium tattooing associated with a system of titanium implant and zirconia abutments. In the two cases, the dark pigments were found after the failure of the zirconia abutment. The biopsy results identified the particulate material dispersed within the tissue was titanium [93]. Within the little amount of report and study on the titanium tattooing, it can be assumed that the releasing of metal particle into gingiva tissue can lead to the formation of the dark color pigmentation, which has the considerable influence on the esthetics, and this phenomenon seems to be related to the fracture or loosening of restoration abutment, when a large amount of metal can be released at a same time. With the development of titanium dental implant in the present, the titanium tattooing is a significant topic that needs more report and study due to its influence on the success of dental implant.

Titanium is known to yield fewer allergic reactions than other metals such as nickel and palladium. However, as mentioned in the “Results” section, titanium allergy symptoms have been reported in some cases [14, 15, 67]. These symptoms can occur systemically with inflammation of the mouth, erythema, etc. Therefore, any history or suspicion of a titanium allergy would be considered prior to dental implant installation. As patients with titanium allergies are allergic to other metals, confirmation of a metal allergic response through a verification of medical history is important; these patients could be required to undergo patch testing prior to dental implant procedures. Patch testing is primarily used to test for allergic reactions to titanium and other metals. In the case of a titanium allergy, there are cases in which detection is not available in blood testing and there are cases in which patients may experience different reactions [69, 94].

Yellow nail syndrome is another important aspect as shown in the “Results” section. In 2011, Berglund and Carlmark [17] reported the first association of titanium with nail accumulation in the nails of patients with yellow nail syndrome. According to several similar reports, patients with titanium dental implants were prevented from developing galvanic interactions with other metal restorations and were reported to have been recovered from their symptoms. There was also a recent prophylactic fluoride treatment among the patients with installed implants. This was due to the corrosion of titanium, which we have seen above, and it was related to yellow nail syndrome in that symptoms recovered when exposure was removed. Additionally, patients with yellow nail syndrome with titanium implants were reported and demonstrated the possibility of relevance. This demonstrated the possibility of systemic disease due to titanium implants and a source of implant failure. It was possible that the corrosion of titanium may have systemic effects rather than only to the surrounding tissues. In this regard, additional attention should be paid to the dental fields as well; diagnosis and treatment of the disease should be performed when similar symptoms occur.

The next issue involves looking at the potential threats of titanium. Titanium is currently used in a wide range of fields. As such, there are many studies with regard to environment safety in the field and basic science. Such titanium is primarily used as titanium oxide nanoparticles. Animal experiments, especially in rodents, are being conducted to study the effects of titanium oxide nanoparticles on the human body. In these experiments, titanium was overdosed in rats and the distribution of titanium in the liver, spleen, bone marrow, lungs, brain, and kidneys and titanium-related problems were found in each tissue [32, 33, 90]. In particular, lung problems, cytotoxic reactions, inflammation reactions, fibrosis, and tumors were observed. Current reports showed the possibility that particle accumulation and ions due to titanium corrosion could be a potential systemic risk. This could be seen in the report that abrasive particles of orthopedic prosthesis were observed in the bone marrow of other tissues [44]. Additionally, there were concerns about the potential of these nanoparticles exhibiting neurotoxicity. There was a report that nanoparticles passed through the BBB, and studies of the effects of these particles on the CNS have been conducted [91]. In the rat experiment mentioned above, titanium was observed to be distributed in the CNS, which was of necessary concern. Although the effects on the human body are not yet known, it is necessary to pay attention to the potential hazards as shown in the animal testing results.

Potential dangers from such an accumulation of titanium include yellow nail syndrome. We consider this as a possible side effect of titanium dental implants as well. In addition to this, it has been reported that patients with periodontal treatment medicines or processed foods coated with titanium, as well as implants, have developed symptoms of yellow nail syndrome [17, 71]. Titanium can accumulate in the body through various pathways, and implant erosion can be an additional pathway; therefore, dentists and other specialists also need to be concerned with these potential risks.

Alternative methods of implant materials are under investigation for the risk of titanium. Typically, these include zirconia and polyetheretherketone (PEEK) dental implants. These materials are considered to be alternatives to the hypersensitivity of titanium and are aimed at improving esthetics. Currently, zirconia is a clinically applied material for dental implants. There have been many studies in the past, and improvements in physical properties, osseointegration, and clinical application have been made [95–97]. These clinical applications have been made, and there have been reports of clinical prognosis after use [95, 96]. Pierall et al. [98] investigated about 347 cases and reported a 95.6% survival rate after a 1-year follow-up in 2016. Marginal bone loss was shown to be around 0.79 mm during the follow-up. Although there is still insufficient data for a long-term follow-up, the result is still somewhat successful.

In the case of PEEK implants, they are currently being studied and are not yet clinically useful. PEEK implants possess a similar elastic modulus to that of bone and have the advantage of imparting less stress on bone than other materials, including titanium, which have a high rigidity. Currently, studies are being performed including animal experiments, and studies are underway to improve their physical properties and osseointegration [97]. However, there is still a lack of physical properties and osseointegration capacity to be clinically applied. These alternative materials are still lacking in research and are not yet clinically applicable to PEEK implants. Currently, clinically applied zirconia implants are limited to clinical applications due to research limitations and long-term data compared with titanium-based implants; the currently commercialized systems are a one-piece system. Until recently, there has not been a system that could completely replace titanium dental implants. Current titanium dental implants have a high success rate except in some cases and will continue to have clinically successful results over a long period of time now that they are being used universally. However, attention must be placed with regard to the hazards of titanium dental implants that are so commonly used. Clinicians should be able to look at the implications of materials in terms of both biological and mechanical aspects when implants fail. It is also important to consider allergic reactions and yellow nail syndrome, which can occur when using titanium dental implants. Clinicians should be aware that, and therefore, it is necessary to explain that these symptoms can occur in any patients when implants are installed. Preventive measures should be considered, and when symptoms occur, patients should be diagnosed accurately and managed appropriately.

## Conclusion

Titanium is used in many fields in addition to being used in dental implants. As the use of titanium increases, concerns over safety are increasing as well. In recent years, studies with regard to titanium toxicity have been on the rise. Although they have mainly been focused on environmental and basic fields, studies are now expanding into the medical field. Thus, there is a need for interest with regard to titanium safety and dangers in the field of dentistry. Titanium dental implants can cause corrosion and wear. Particles and ions of titanium and titanium alloy components due to corrosion and wear can be deposited in surrounding tissues, and inflammation reactions can occur. The accumulation of titanium ions and particles can occur systemically as well as in the surrounding tissues, which can lead to toxic reactions in other tissues including yellow nail syndrome. Additionally, there are cases where the metal material is hypersensitive. Currently, zirconia implants are considered to be an alternative; however, there are still limitations due to a lack of long-term clinical data. Within the limitation of this study, it was suggested that we should be aware of the rare problems of titanium toxicity.
